# Metallothionein 1M suppresses tumorigenesis in hepatocellular carcinoma

**DOI:** 10.18632/oncotarget.16521

**Published:** 2017-03-23

**Authors:** Cheng-Lin Fu, Bing Pan, Ju-Hua Pan, Mei-Fu Gan

**Affiliations:** ^1^ Department of Pathology, The First Hospital of Taizhou, Wenzhou Medical University, Taizhou 318020, China; ^2^ Department of Pathology, Taizhou Hospital, Wenzhou Medical University, Linhai 317000, China

**Keywords:** hepatocellular carcinoma, metallothionein 1M, MT1M, tumor marker

## Abstract

Members of the metallothionein (MT) family are involved in metal detoxifcation and in the protection of cells against certain electrophilic carcinogens. In present study, it was found that MT1M was downregulated in more than 77.1% (91/118) of hepatocellular carcinoma (HCC) tissues compared with adjacent non-tumor tissues. Furthermore, overexpression of MT1M inhibited cell viability, colony formation, cell migration and invasion in HCC cell lines and tumor cell growth in xenograft nude mice, and activated cell apoptosis in HCC cell lines. In addition, immunohistochemistry analysis showed MT1M was negative or weak staining in tumor tissues but moderate or strong staining in adjacent non-tumor tissues. The sensitivity and specificity of MT1M for HCC diagnosis were 76.27% and 89.83%, respectively. In conclusion, MT1M was identified as a potential tumor marker for HCC and may serve as a useful therapeutic agent for HCC gene therapy.

## INTRODUCTION

Hepatocellular carcinoma (HCC) is the fifth most common cancer across the world [1]. Each year more than 500,000 new patients are diagnosed with HCC in the world [2]. However, early diagnosis of HCC is complicated by the coexistence of inflammation and cirrhosis. Thus, novel biomarkers for HCC early diagnosis are required [3]. Over the last decade, numerous novel biomarkers were identified due to the advances in genomics and proteomics techniques. These biomarkers are being developed not only for use of HCC diagnosis, but also in prediction of patient and treatment outcomes and individualization of therapy [4–6]. Some of them are more promising, such as glypican-3(GPC3) [7], osteopontin(OPN) [8], Des-γ-carboxyprothrombin(DCP) [9], Golgi protein-73(GP73) [10] and microRNAs (miR-122 and miR-21, et al.) [11, 12].

Members of the metallothionein (MT) family are small cysteine-rich proteins, which were previously reported mostly associated with cellular metabolism of metal ions, especially zinc [13]. Moreover, increasing evidence suggests MTs play a role in the regulation of cellular or pathological processes including various cancers [14]. Recently, dramatic decrease of MT family in HCC has been reported by several research groups [15–17]. In addition, MT1M promoter methylation was reported as biomarkers for HCC or breast cancer [18, 19].

In the present study, we investigated the roles of MT1M in HCC tumorigenesis and further evaluated the usefulness of MT1M as a biomarker for the diagnosis of HCC.

## RESULTS

### MT1M was downregulated in HCC tumor tissues

**Figure 1:**
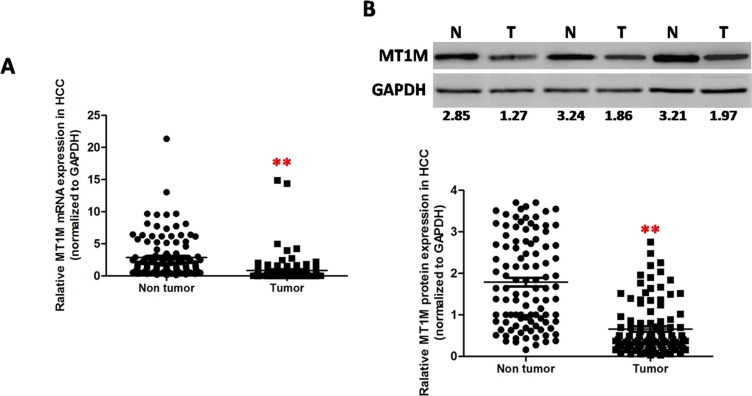
MT1M was downregulated in HCC tumor tissues (**A**) Downregulated expression of MT1M in HCC tumor tissues (T) compared with adjacent non-tumor tissues (N) (*n* = 118,*p < 0.01, **p < 0.001) by quantitative RT-PCR. (**B**) Downregulated expression of MT1M in HCC tumor tissues (T) compared with adjacent non-tumor tissues (N) by Western blot. Upper panel: representative data of 3 paired HCC tissues; Lower panel: the summary data of 118 paired HCC tissues (*p < 0.01, **p < 0.001).

Totally, 2153 deregulated genes were identified, including 1065 upregulated genes and 1088 downregulated genes. Forty one deregulated mRNA (20 upregulation and 21 downregulation) showed > 10 fold changes and most of these deregulated genes have been reported in the HCC (Supplementary Table 1) [21–29]. Interesting, 3 MT superfamily numbers, MT1M, MT1G and MT1P2 were significantly down-regulated in the HCC tumor tissues. To validate the downregulation of MT1M in HCC, qRT-PCR and Western blot were performed in 118 pairs of HCC tissues. In total, it was found that MT1M was downregulated in 91 (77.2%) samples of HCC tumors (Figure 1A and 1B).

### MT1M inhibits cell growth in cell lines and xenograft model

**Figure 2:**
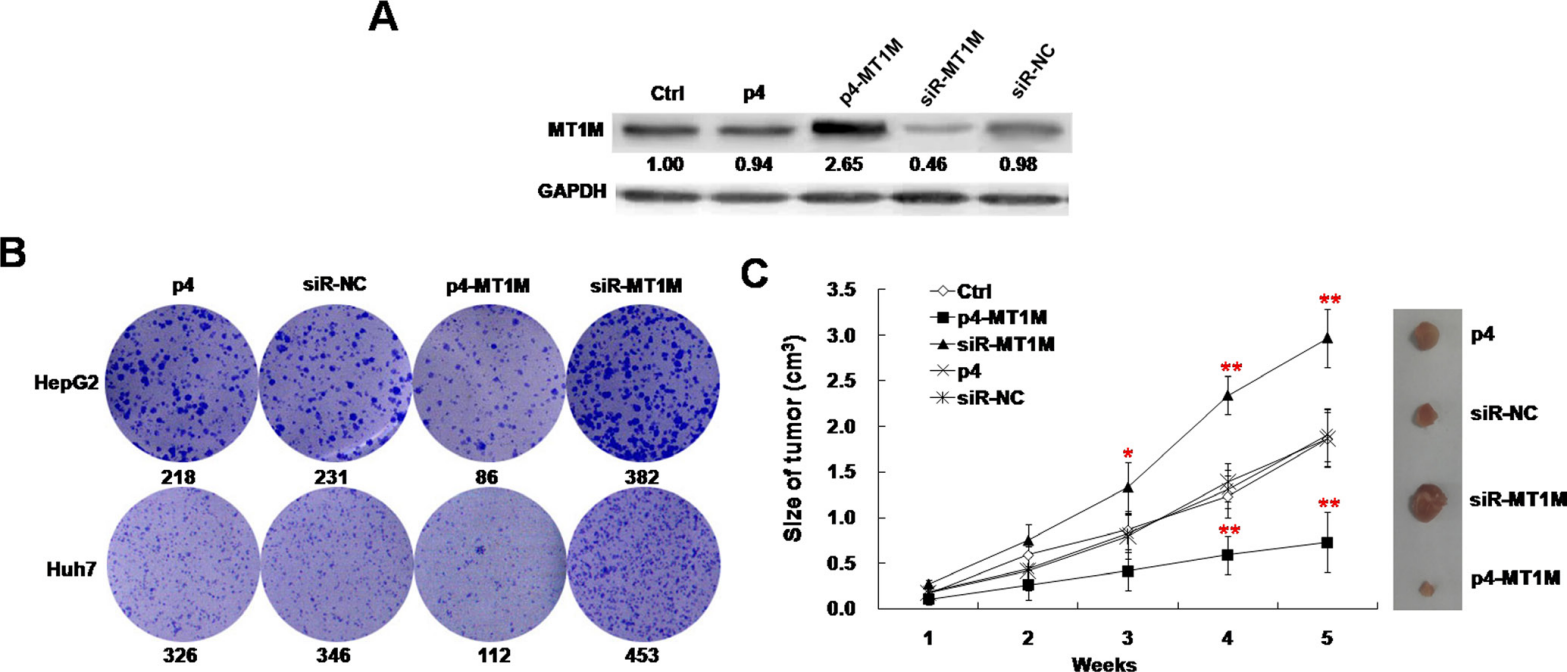
MT1M suppressed tumor growth *in vitro* and *in vivo* (**A**) MT1M overexpression and knockdown in HepG2 cells. The expression of MT1M was analyzed in HepG2 cells transfected with p4-MT1M, siR-MT1M or p4 and siR-NC by Western blot. GAPDH was used as an internal control. (**B**) The effect of MT1M on colony formation of HCC cell lines. (**C**) The effect of MT1M on tumor growth in xenograft nude mice. *p < 0.01, **p < 0.001.

To explore its role in tumorigenicity, p4-MT1M and siR-MT1M were transfected into HepG2 and Huh7. The expressions of MT1M with over-expression or knockdown transfectants were confirmed by Western blot analysis (Figure 2A).

To investigate the effect of MT1M on the growth of HCC cells, the colony formation assay was performed in HCC cells transfected with p4-MT1M or siR-MT1M. It was found that p4-MT1M transfected HCC cells had fewer colonies (86 or 112 colonies), siR-MT1M transfected cells had more colonies (382 or 453 colonies), but p4 transfected cells (218 or 326 colonies) and siR-NC transfected cells (231 or 346 colonies) had similar number of colonies (Figure 2B). In further, xenograft nude mouse model was used to investigate the role of MT1M *in vivo*. Growth rates of tumor showed a significant decrease at 4th and 5th week in p4-MT1M expressing group and a significant increase at the 3rd, 4th and 5th week in siR-MT1M expressing group, whereas no significance in siR-NC transfected or nontransfected HepG2 cells groups (Figure 2C). Consistent with the above findings, tumors induced by p4-MT1M transfected HepG2 cells showed significantly smaller mean tumor volume, but tumors induced by siR-MT1M transfected HepG2 cells showed significantly larger mean tumor volume, than tumors induced by siR-NC transfected or nontransfected HepG2 cells (Figure 2C). These results indicated that MT1M significantly inhibits HCC tumorigenesis *in vivo*.

### MT1M inhibits cell viability by inducing cell cycle arrest and enhancing cell apoptosis

**Figure 3:**
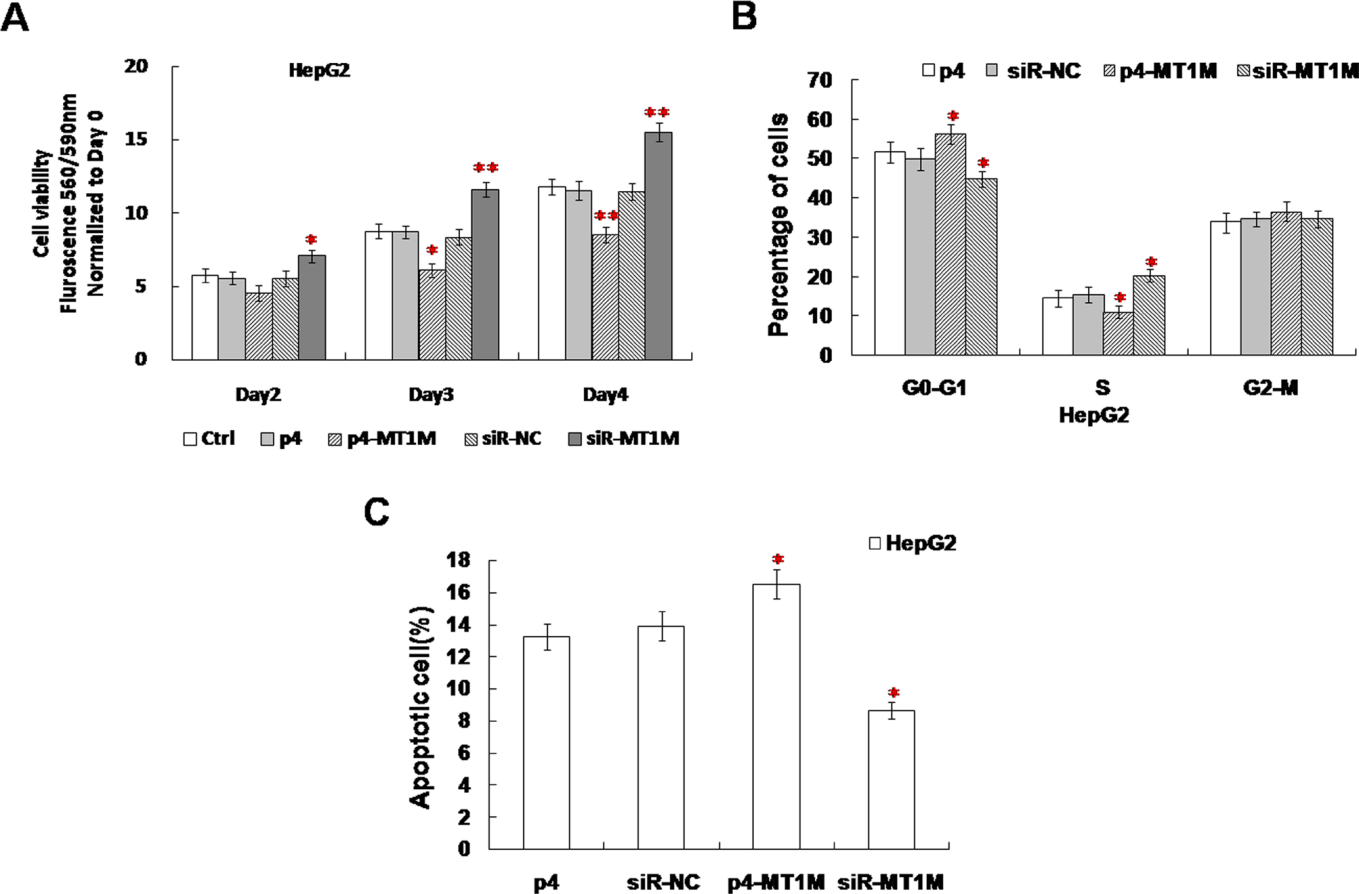
MT1M suppressed cell viability, cell cycle and apoptosis (**A**) The effect of MT1M on cell viability. (**B**) The effect of MT1M on cell cycle. (**C**) The effect of MT1M on cell apoptosis. Representative results were from HepG2 cells transfected with p4, siR-NC, p4-MT1M or siR-MT1M respectively. Column, mean of three independent experiments; bars, SD; *p < 0.01; **p < 0.001.

In cell viability assay, from 2~3 day, the viability of p4-MT1M transfected cells decreased significantly, but the viability of siR-MT1M transfected cells with increased significantly (Figure 3A). These results indicate MT1M could inhibit the growth of HCC cells.

Cell cycle analysis showed that much more p4-MT1M transfected cells were in G0-G1 phase and fewer siR-MT1M transfected cells were in G0-G1 phase (Figure 3B). There is no significance in siR-NC transfected or nontransfected cells (Figure 3B). In addition, the apoptotic cells in p4-MT1M transfected cells were 36.4% more than that in p4 transfected cells (Figure 3C). In siR-MT1M transfected cells, the apoptotic cells were 21.5% less than that in siR-NC transfected cells (Figure 3C). These results indicated MT1M could inhibit HCC cell growth by arresting cell cycle from G0/G1 to S phase and enhancing apoptosis.

**Figure 4:**
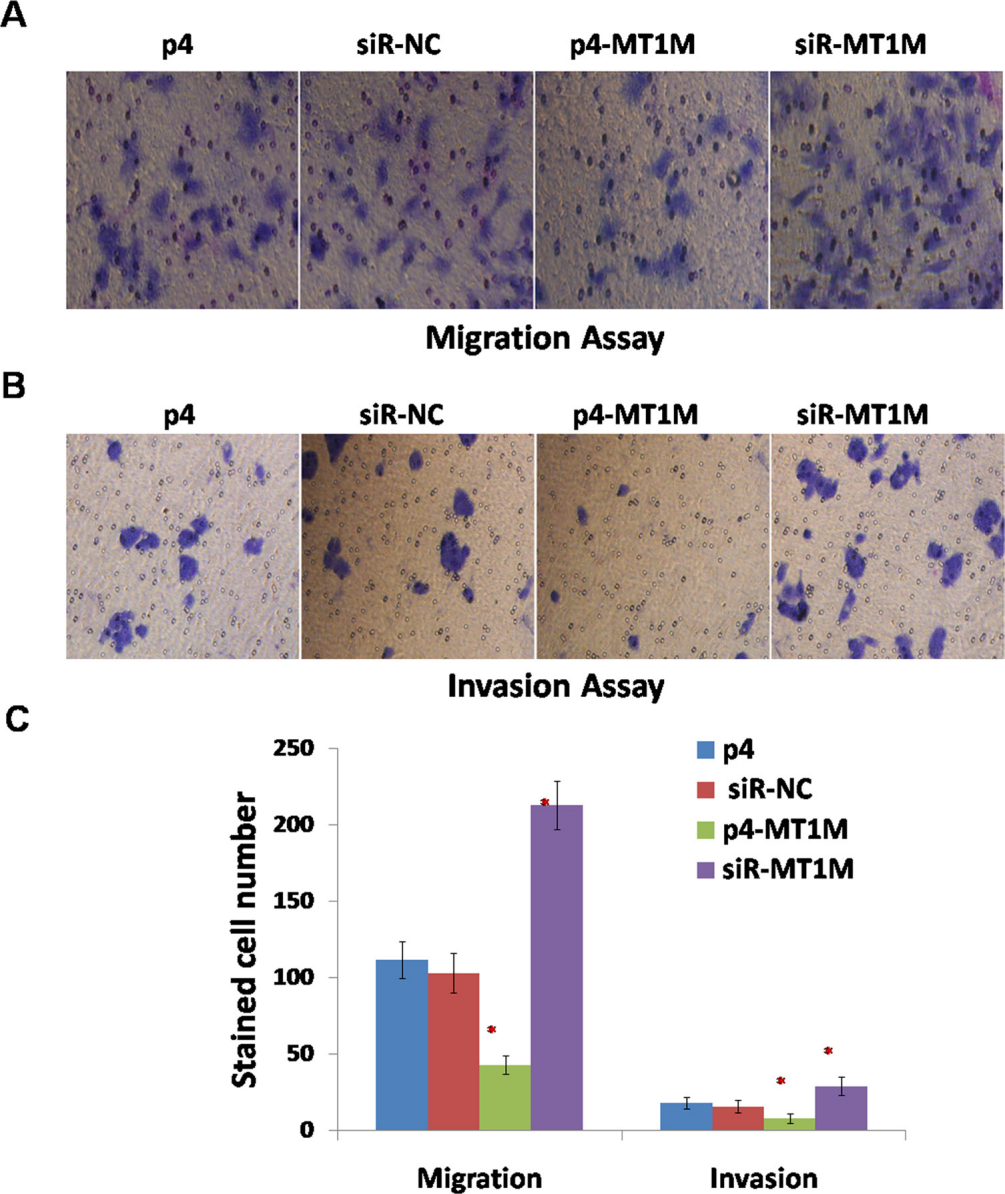
MT1M suppressed cell migration and invasion (**A**) The effect of MT1M on cell migration. (**B**) The effect of MT1M on cell invasion. (**C**) Summary of data in A&B. Representative results were from HepG2 cells transfected with p4, siR-NC, p4-MT1M or siR-MT1M respectively. Column, mean of three independent experiments; bars, SD; *p < 0.01; **p < 0.001.

To verify the effects of MT1M on HCC tumorigenesis in further, the migration of HepG2 cells were investigated. For p4-MT1M transfected cells, there are less cells that migrated to the lower surface of the filter, but for siR-MT1M transfected cells, there are more cells that migrated to the lower surface of the filter, compared with p4 or siR-NC transfected cells (Figure 4A and 4C). Moreover, to substantiate this observation, a Matrigel invasion assay was carried out to determine the effect of MT1M on the invasion of HepG2 cells. Similar results were observed (Figure 4B and 4C). These results indicated MT1M might inhibit cell migration and invasion of HCC.

### Downregulated MT1M is associated with HCC pathological progress

**Figure 5:**
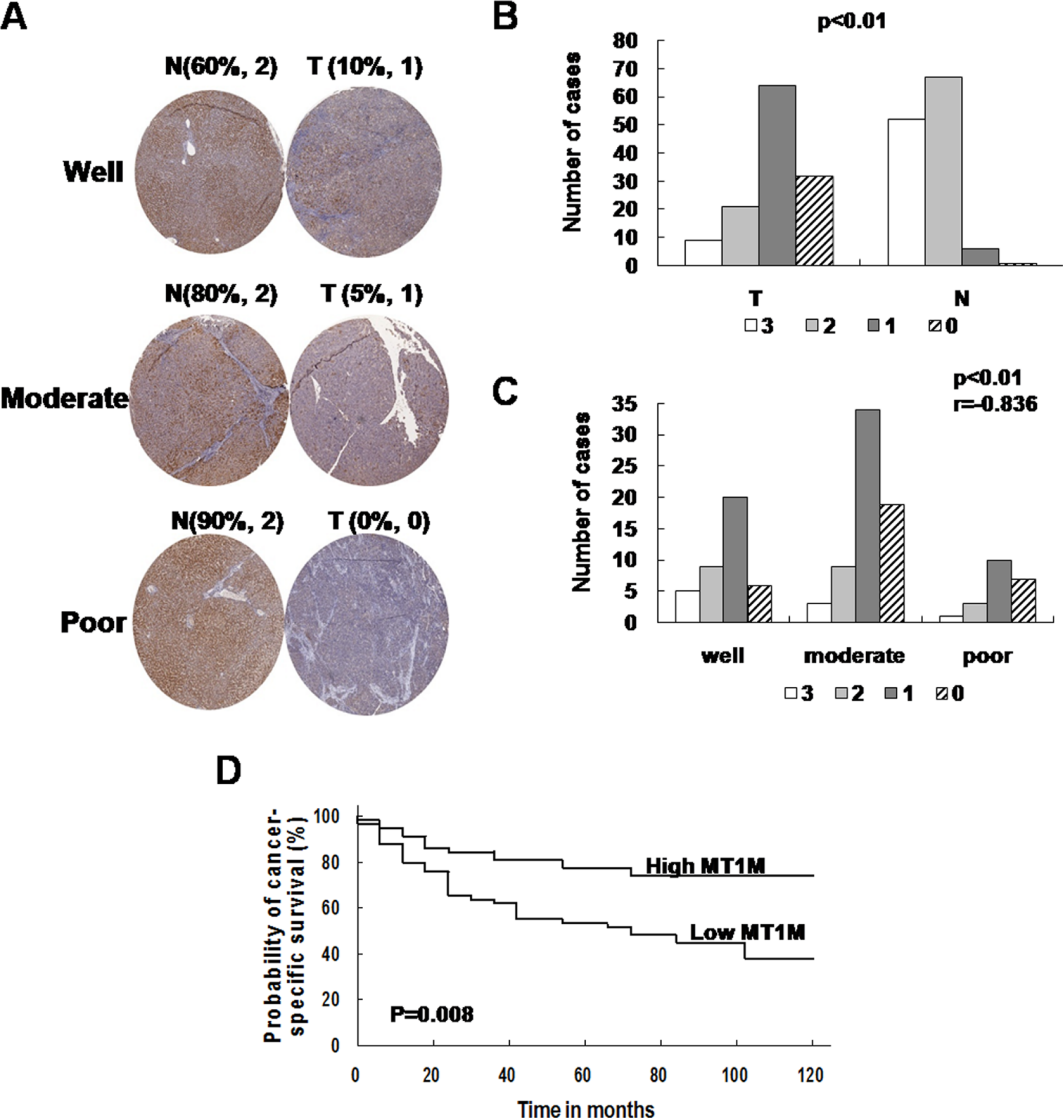
MT1M was associated with cell differentiation and pathology of HCC (**A**) MT1M was stained in HCC tissue arrays. Strong or moderate staining of MT1M in the cytoplasm of HCC non-tumor tissues but compared with negative or weak staining in tumor tissues was observed. Well: well differentiated HCC; Moderate: moderately differentiated HCC; Poor: poorly differentiated HCC. (**B**) The summary data of HCC tissue arrays. (**C**) MT1M expression level correlates with tumor differentiation. r: correlation coefficient. (**D**) Probability of cancer specific survival by levels of MT1M expression in HCC.

To evaluate the usefulness of MT1M for HCC diagnosis, HCC tissue arrays (Supplementary Table 2) were used. As the representative results, MT1M was strong stained in the cytoplasm of non-tumor cells, but in tumor cells, staining was weak or negative (Figure 5A). Furthermore, MT1M expression was weak or negative in most HCC tumor tissues (90 of 118 cases). However, MT1M expression was moderate or strong (score 2~3) in most non-tumor tissues (106 of 118 cases) (Figure 5B, Table 1). The sensitivity of MT1M for HCC diagnosis was 76.27% and the specificity was 89.83% (Table 1). It was indicated that MT1M might be a potential biomarker for HCC early diagnosis.

**Table 1:** Immunohistochemical analysis from HCC tissue array

A								
	Tumor				Nontumor			
Expression	Well	Moderate	Poor	Total	normal	Hepatitis	Cirrhosis	Total
3	0	0	0	0	5	28	10	38
2	0	3	5	28	13	19	8	68
1	6	29	37	62	20	14	1	12
0	5	11	22	28	0	0	0	0
Total	11	43	64	118	38	61	19	118

B						
Markets	Tumor		Nontumor		Sensitivity	Specificity
	+	−	+	−	%	%
MT1M < 2	90	28	12	106	76.27	89.83

The expression level of MT1M reverse correlates with tumor differentiation in HCC (r = −0.836, *P* < 0.01) (Figure 5C). Furthermore, patients whose primary tumors without MT1M downregulation have a trend of well survival (Figure 5D). The mean survival of low MT1M expression group was 68.4 months (poor survival group, *n* = 89), whereas the mean survival of high MT1M expression group was 99.8 months (good survival group, *n* = 29). No statistical correlation was observed between MT1M expression and other clinic pathological features (sex, age, tumor stage, et al.) (data not shown). It was indicated that MT1M might be associated with tumor differentiation and immunostaining of MT1M might be usefulness to HCC diagnosis.

## DISCUSSION

MTs consisted of at least 4 main isoforms (MT-1, MT-2, MT-3 and MT-4) in mammals [30]. MT1 and MT2 are frequently downregulated in HCC [31, 32]. Ding J, et al. reported that low MT1M expression correlated with tumor recurrence and disease-free survival [33]. Dong X, et al. reported that MT1M was downregulated in HCC tumor tissues significantly and miR-24-3p/MT1M pathway may contribute to the initiation and progression of HCC [34]. Ji XF, et al. reported that MT1M promoter methylation was significantly increased in HCC group and was positively correlated with HCC tumor size [35]. Mao J, et al. reported that MT1M is often present at lower levels and its promoter is often with methylation in HCC tumors and cells [20]. All of these findings are consistent with the results of the present study.

Downregulation of MT1M in HCC tumor tissues was reported well, however, the mechanism of MT1M effect needs further verification by multi-groups, and the application of MT1M as HCC biomarker for clinical diagnosis needs evaluation. In the present study, MT1M overexpression significantly inhibited cell growth by inducing cell cycle arrest and enhancing apoptosis of HCC cells, while MT1M knockdown obtained the reverse results. These findings indicate that MT1M suppresses HCC tumorigenesis. We further investigated the inhibitory role of MT1M in cell migration and invasion. These results strengthen the hypothesis that MT1M served as a tumor suppressor.

Moreover, we further investigated the expression of MT1M in HCC tissue chip by immunohistochemistry analysis and evaluated the potential usefulness for HCC diagnosis. Consistent with results of Western blot, MT1M was significantly downregulated in HCC tumors and MT1M expression was associated with cell differentiation significantly. Well differentiated tumors were profoundly strong for MT1M expression, but MT1M expression was negative in the most moderately and poorly differentiated tumors. Thus, MT1M expression might be associated with the tumor cell differentiation. Furthermore, the sensitivity and specificity of MT1M for HCC diagnosis is even similar to those of GPC-3 reported [36]. Thus, MT1M might be diagnostically useful HCC screening. Further study was needed to validate the accuracy of MT1M for HCC diagnosis in much larger populations.

In conclusion, it were found that: 1) downregulated MT1M in HCC tumors was a potential tumor marker; 2) MT1M suppressed HCC tumorigenesis possibly by inducing cell cycle arrest, enhancing apoptosis and inhibiting cell migration and invasion. These findings provide evidence that MT1M might be a tumor suppressor and potential marker for HCC gene therapy and early diagnosis.

## MATERIALS AND METHODS

### HCC tissue specimens and cell lines

118 pairs of human HCC tumor and adjacent non-tumor tissues were obtained from surgical specimens immediately after resection from patients in the Taizhou Hospital, Zhejiang Province, China. The samples were frozen in liquid nitrogen and stored at −80°C until use. Among these samples, three pairs were used for Human Gene Expression Array analysis and all of them were used for quantitative real-time PCR (qRT–PCR) and Western blot analysis. Clinical and pathological information was extracted from the patients’ medical charts and pathology reports (Table 2). Informed consent for tissue donation (for research purposes) was obtained from the patients before tissue collection and the protocol was approved by the Institutional Review Board of Taizhou Hospital of Zhejiang Province.

**Table 2:** The data of patients

A				
Patients	Age	Tumor size	Tumor grade	TNM stage
P1	68	9.2	G3	III
P2	62	6.5	G3	II
P3	65	8.4	G3	II

B	
Variable (*n* = 118)	Value
**Age** (years)	56.8 ± 12.4
**Gender** (male)	86(72.8%)
Tumor size* (cm)	6.73
**Tumor grade**	
Well-differentiated (G1-2)	36
Moderately-differentiated (G3)	42
Poorly-differentiated (G4)	40
TNM stage**	
I	30
II	48
III	40
IV	0

* Diameter of the biggest nodule.

** TNM: tumor-node-metastasis.

Human HCC cells lines HepG2 and Huh7 were employed in this study, maintained in DMEM medium with 10% fetal bovine serum (Invitrogen, Carlsbad, CA, USA) at 37°C in a humidified atmosphere containing 5% CO_2_.

### Gene expression profiling

Total RNA was isolated using TRI Reagent combined with the RNeasy Tissue kit protocol (Qiagen, Valencia, CA) according to the manufacturer's recommendations. To identified differential genes in HCC tumor tissue, the PrimeView™ Human Gene Expression Array (Affymetrix, Santa Clara, CA) was performed in 3 pairs of HCC tumor and adjacent non-tumor tissues according to the manufacture's protocol. A total of 750 ng of labeled complementary RNAs were hybridized to arrays and then imaged using Affymetrix Fluidics Station FS450 and scanned with GeneChip Scanner 3000 7G according to manufacturer's instructions. Raw signals of the arrays were processed using Affymetrix Power Tools. Data quality was assessed based on the positive and negative control probes on each array as well as by inspection of the distributions of probe intensities. Data was normalized using the quantile normalization method. A moderated *t*-test implemented in the limma library of bioconductor was applied to test differential expression, and a false discovery rate (FDR) adjustment of the *p*-value was performed to correct for multiple testing. Probes were considered significantly different if the adjusted *p*-value was less than 0.05 and the fold change difference between groups was at least 2.

### Immunohistochemistry

Tissue arrays (Super Chip, Shanghai, China) were used for immunohistochemistry, which contained 126 paired HCC liver tissues (Supplementary Table 2). Tissues were deparaffinized in xylene and rehydrated by reducing the concentration of ethanol (100% and 70%, 5 minutes each). Antigens were unmasked with microwave irradiation for 5 minutes in pH 6.0 citric buffer three times. The slides were incubated with MT1M antibodies (1:100 dilution) at 4°C overnight, followed by incubation with HRP-conjugated goat anti-rabbit (1:1000 dilution, KPL) at room temperature for 60 minutes. Finally, 3, 3′-diaminobenzidine tetrahydrochloride (DAB) was used for signal development, and 20% hematoxylin was used for counterstaining. The slides were dehydrated, cleared and evaluated by three pathologists. The expression level of MT1M was scored as 0 (negative), 1 (weak), 2 (moderate), or 3 (strong) by the staining intensity of the protein. ROC curve between HCC tumor and adjacent non-tumor tissues was plotted and the sensitivity and the specificity of MT1M were determined.

### Construction of plasmids

The full length of MT1M (Genbank accession no. NM_176870.2) was amplified with the primers 5′- TC CGGGTGGGCCTAGCAGTCG-3′ (forward) and 5′-GGC ACAGCAGCTGCAGTTCTC-3′ (reverse). The PCR product was cloned into mammalian expression vector pcDNA^TM^4/*myc-his* (Invitrogen), confirmed by sequencing analysis and termed as p4-MT1M.

### Transfection

Transfection was performed with FuGene HD transfection reagent (Roche) according to the manufacturer's protocol. In brief, 2 × 10^4^ HepG2 or Huh7 cells in 24-well plate were transfected with indicated plasmids or siRNAs (GenePharma) and collected for assay 24~48 hours after transfection.

### Western blot

Protein extracts from HCC tissues or HepG2 cells were prepared by a modified RIPA buffer with 0.5% sodium dodecyl sulfate (SDS) and proteinase inhibitor cocktail (Complete mini, Roche, Indianapolis, IN, USA). Fifty micrograms of protein were electrophoresed in 10% SDS-PAGE mini gels and transferred onto PVDF membranes (Immobilon P^−SQ^, Millipore, Billerica, MA, USA). After blocking with 5% nonfat milk, the membranes were incubated with MT1M antibody (1:1000, Sigma-Aldrich, St. Louis, MO, USA) or GAPDH antibody (1:5000, Epitomics Inc., Burlingame, CA, USA at 4°C overnight, followed by incubation with HRP-conjugated goat anti-rabbit or goat anti-mouse antibody (1:10000 dilution, KPL, Gaithersburg, MA,USA) for 1 hour at room temperature. Finally, signals were developed with Super Signal West Pico chemoluminescent substrate (Pierce, Rockford, IL, USA), visualized by the Gene Gnome HR Image Capture System (Syngene, Frederick, MD, USA) and analyzed by Gene tools (Syngene).

### Cell viability assay

Transfectants (HepG2 and Huh7 cells) were maintained in fresh 96-well plate with DMEM containing 10% FBS for 7 days. Cells were tested for proliferation per 24 hours using Cell Titer-Blue cell viability assay (Promega Corporation) according to the manufacturer's instructions and the fluorescence value was recorded by multi-plate reader (Synergy 2).

### Colony formation assay

Transfectants (HepG2 and Huh7 cells) were selected using G418 (800 μg/ml) for 2–3 weeks and then cell colonies were fixed with 20% methanol and stained with 0.1% coomassie brilliant blue R250 at room temperature for 15 min. The total number of colonies in each plate from three independent transfections was counted by ELIspot Bioreader 5000 (BIO-SYS, Karben, GE).

### Cell cycle assay

Transfected HepG2 and Huh7cells were harvested, washed once in phosphate buffer saline (PBS), and fixed in 70% ethanol at 4°C overnight. Staining for DNA content was performed with 50 mg/mL propidium iodide and 1 mg/mL RNase A at room temperature for 30 minutes. Populations in G0-G1, S, and G2-M phase were measured by Cell Lab Quanta SC flow cytometry (Beckman Coulter, Fullerton, CA, USA) and the data were analyzed by FlowJo v7.6 Software.

### Apoptosis assay

Transfected HepG2 and Huh7cells were harvested, washed once in phosphate buffer saline (PBS), resuspended and incubated with FITC-Annexin V (Promega Corporation) for 15 minutes at 4°C in the dark, according to the manufacturer's instructions. After staining, the cells were incubated with propidium iodide for 5 minutes at 4°C in the dark and then analyzed by Cell Lab Quanta SC flow cytometry and the data were analyzed by FlowJo v7.6 Software.

### Tumor xenograft assay

Male BALB/c nude mice (5 to 6 weeks of age) were obtained from Zhejiang Experimental Animal Center (Zhejiang, China) and acclimated to laboratory conditions 1 week before tumor implantation. For *in vivo* tumorigenicity assay, all pyrimidine nucleotides in the MT1M siRNA or NC siRNA were substituted by their 2′-O-methyl analogues to improve RNA stability. HepG2 cells (1 × 10^5^) stably expressing MT1M or transfected with MT1M siRNA were suspended in 100 uL PBS and then injected s.c. into the left side of the posterior flank of 6 BALB/c nude mice, respectively. NC siRNA or pcDNA3.1 transfected HepG2 cells (1 × 10^5^) were injected subcutaneously into the right side of same 12 mice. Tumors were measured every week and the volumes were calculated using the formula for hemi-ellipsoids: V = length (cm) × width(cm) × height (cm) × 0.5236. After 5 weeks, the mice were sacrificed and the tumors were dissected for further examination. Animal handling and experimental procedures were approved by the Animal Experiments Ethics Committee of Taizhou Hospital of Zhejiang Province.

### Cell migration and invasion assays

For the cell migration assay, 2 × 10^5^ HepG2 or Huh7 cells transfected with p4-MT1M or siR-MT1M were seeded in the upper chamber of transwell units (Corning, NY, USA) with 8μm pore size polycarbonate filter under serum free condition. The lower chamber was filled with 500 μL DMEM containing 10% FBS. After incubation for 24h, cells on the upper surface of the filter were completely removed by wiping with a cotton swab. Then the filters were fixed with 4% paraformaldehyde and stained with 0.1% coomassie brilliant blue R250 for 20 min. The number of cells that migrated through the pores to the lower surface of the filter was counted and analyzed with a digital microscope system (IX81; Olympus). Triplicate samples were acquired and the data were expressed as the average cell number of 5 fields. For the cell invasion assay, the similar protocol of cell migration assay was used except that the transwell units were pre-coated with 200μg/ml Matrigel (BD Biosciences, San Jose, CA, USA) and incubated overnight. Cells that had invaded the Matrigel and reached the lower surface of the filter were counted.

### Statistical analysis

All experiments were performed at least three times, and data are presented as mean ± SD. Statistical analyses were carried out with SPSS17.0. Comparisons were made by using a two-tailed t test or one-way ANOVA for experiments with more than two subgroups. Correlation analysis was made by using Spearman correlation coefficient. *P* < 0.01 was considered statistically significant. Association of MT1M expression with cancer specific survival rate was analyzed using the Kaplan-Meier method.

## SUPPLEMENTARY MATERIALS TABLES


