# Supplementary material for: Metallothionein 1M suppresses tumorigenesis in hepatocellular carcinoma
Source: Oncotarget. 2017 Mar 23;8(20):33037–46. doi: 10.18632/oncotarget.16521 (PMC5464848; doi:10.18632/oncotarget.16521)
Supplement: Supplementary file 1 [file oncotarget-08-33037-s001.pdf]

# Metallothionein 1M suppresses tumorigenesis in hepatocellular carcinoma

## Supplementary Materials

### Supplementary Table 1: Deregulated mRNAs in HCC tumor tissues

| Symbol | Description | Fold change | *p*-value |
|---|---|---|---|
| **Up-regulated mRNAs** | | | |
| PGC | progastricsin (pepsinogen C) | 38.27004827 | 0.0014 |
| AFP | alpha-fetoprotein | 34.92471892 | 0.0028 |
| AKR1B10 | aldo-keto reductase family 1, member B10 (aldose reductase) | 34.74267855 | 0.0033 |
| GPC3 | glypican 3 | 33.36178168 | 0.0028 |
| REG3A | regenerating islet-derived 3 alpha | 27.11723945 | 0.0019 |
| SPP1 | secreted phosphoprotein 1 (osteopontin, early T-lymphocyte activation 1) | 25.96742678 | 0.0017 |
| SQLE | squalene epoxidase | 23.84278995 | 0.0027 |
| FADS2 | fatty acid desaturase 2 | 21.89356206 | 0.0025 |
| ACSL4 | acyl-CoA synthetase long-chain family member 4 | 16.80832764 | 0.0041 |
| TOP2A | topoisomerase (DNA) II alpha 170 kDa | 15.47422559 | 0.0051 |
| MKI67 | antigen identified by monoclonal antibody Ki-67 | 14.74324575 | 0.0035 |
| NQO1 | NAD(P)H dehydrogenase, quinone 1 | 14.71561691 | 0.0022 |
| CENPF | centromere protein F, 350/400 ka (mitosin) | 14.37592617 | 0.0038 |
| ANLN | anillin, actin binding protein | 13.34102265 | 0.0019 |
| PROM1 | prominin 1 | 10.79763216 | 0.0031 |
| SPINK1 | serine peptidase inhibitor, Kazal type 1 | 10.7974104 | 0.0054 |
| ***Down-regulated miRNAs*** | | | |
| TAT | tyrosine aminotransferase | 0.015473228 | 0.0017 |
| JDP2 | Jun dimerization protein 2 | 0.016423237 | 0.0031 |
| HSD17B13 | hydroxysteroid (17-beta) dehydrogenase 13 | 0.017354256 | 0.0042 |
| CYP2B6 | cytochrome P450, family 2, subfamily B, polypeptide 6 | 0.023546577 | 0.0046 |
| PCK1 | phosphoenolpyruvate carboxykinase 1 (soluble) | 0.027342216 | 0.0038 |
| GLYAT | glycine-N-acyltransferase | 0.033147342 | 0.0043 |
| ABCA8 | ATP-binding cassette, sub-family A (ABC1), member 8 | 0.044636343 | 0.0017 |
| CYP1A2 | cytochrome P450, family 1, subfamily A, polypeptide 2 | 0.051754747 | 0.0025 |
| GYS2 | glycogen synthase 2 (liver) | 0.056743522 | 0.0026 |
| SLC22A1 | solute carrier family 22 (organic cation transporter), member 1 | 0.058242678 | 0.0017 |
| HPD | 4-hydroxyphenylpyruvate dioxygenase | 0.064676894 | 0.0053 |
| MT1M | metallothionein 1M | 0.069352352 | 0.0051 |
| MT1G | metallothionein 1G | 0.072546578 | 0.0036 |
| FOS | v-fos FBJ murine osteosarcoma viral oncogene homolog | 0.076365768 | 0.0042 |
| MT1P2 | metallothionein 1 pseudogene 2 | 0.083257688 | 0.0028 |

**Supplementary Table 2: Histology of HCC tissues on tissue array**

| | Tumor | | |
|---|---|---|---|
| **Non-tumor** | **Well** | **Moderately** | **Poorly** |
| Normal | 10 | 2 | 0 |
| Hepatitis | 21 | 26 | 0 |
| Cirrhosis | 11 | 37 | 19 |
| Total | 42 | 65 | 19 |
